# Text Reading Fluency and Text Reading Comprehension Do Not Rely on the Same Abilities in University Students With and Without Dyslexia

**DOI:** 10.3389/fpsyg.2022.866543

**Published:** 2022-05-09

**Authors:** Hélène Brèthes, Eddy Cavalli, Ambre Denis-Noël, Jean-Baptiste Melmi, Abdessadek El Ahmadi, Maryse Bianco, Pascale Colé

**Affiliations:** ^1^Aix-Marseille Université, CNRS, Laboratoire de Psychologie Cognitive (UMR 7290, CNRS), Marseille, France; ^2^Brain and Language Research Institute/Institute of Language, Communication and the Brain, Aix-Marseille Université and CNRS, Aix-en-Provence, France; ^3^Laboratoire d’Etude des Mécanismes Cognitifs, Université Lumière Lyon 2, Lyon, France; ^4^Université Côte d’Azur, CNRS, MSHS Sud-Est, CoCoLab, Nice, France; ^5^Aix-Marseille Université, CNRS, Laboratoire de Neurosciences Cognitives (UMR 7291, CNRS), Marseille, France; ^6^Université Grenoble Alpes, LaRAC, Grenoble, France

**Keywords:** adults with dyslexia, reading comprehension, text reading fluency, compensation, reading

## Abstract

Developmental dyslexia is a specific learning condition characterized by severe and persistent difficulties in written word recognition, decoding and spelling that may impair both text reading fluency and text reading comprehension. Despite this, some adults with dyslexia successfully complete their university studies even though graduating from university involves intensive exposure to long and complex texts. This study examined the cognitive skills underlying both text reading comprehension and text reading fluency (TRF) in a sample of 54 university students with dyslexia and 63 university students without dyslexia, based on a set of tests adapted for an adult population, including listening comprehension, word reading, pseudoword reading (i.e., decoding), phonemic awareness, spelling, visual span, reading span, vocabulary, non-verbal reasoning, and general knowledge. The contribution of these skills to text reading fluency and text reading comprehension was examined using stepwise multiplicative linear regression analyses. As far as TRF is concerned, a regression model including word reading, pseudoword reading and spelling best fits the data, while a regression model including listening comprehension, general knowledge and vocabulary best fits the data obtained for text reading comprehension. Overall, these results are discussed in the light of the current literature on adults with dyslexia and both text reading fluency and text reading comprehension.

## Introduction

Developmental dyslexia (hereafter dyslexia), a specific learning disorder which affects 10% of the population is primarily characterized by significant difficulties in written word recognition, slow and inaccurate decoding that may impair reading comprehension, and poor spelling performance (American Psychiatric Association, 2013). Because most symptoms of dyslexia persist in adulthood, it is considered to be a non-transient developmental deficit and its prevalence in adults can therefore be considered to be stable. However, an increasing number of students with dyslexia are entering and graduating from higher education^1^. Some studies have reported that these readers can exhibit text reading comprehension performance comparable to that of adult skilled readers of the same chronological age (Parrila et al., 2007; Deacon et al., 2012; Hebert et al., 2018; Cavalli et al., 2019), despite impairments in decoding and written word recognition (Bruck, 1990, 1992; Pennington et al., 1990; Kemp et al., 2009; Martin et al., 2010; Cavalli et al., 2018). However, according, for example, to the *verbal efficiency hypothesis* (Perfetti, 1985), fluent and efficient written word recognition is a fundamental pre-requisite for achieving good text comprehension (Gough and Tunmer, 1986; Perfetti and Hart, 2002). Consequently, inefficient written word recognition (i.e., slow and inaccurate) is likely to impair reading comprehension (Perfetti, 1985). This does not always appear to be the case for university students with dyslexia and studies (mentioned above) have reported a dissociation between performance on visual word recognition and/or decoding skills and reading comprehension skills (see, for example, Cavalli et al., 2019). Because it is possible that reading comprehension in this population cannot be reliably predicted on the basis of both visual word recognition and decoding skills, it is possible to hypothesize that these readers have probably developed compensatory and/or adaptive mechanisms induced by continued exposure to written texts (Lefly and Pennington, 2000) that allow them to understand a text at the same level as typical readers. Interestingly, it can also be argued that skills associated with the recognition of written words may not provide an adequate basis for estimating reading skills when compared to text reading fluency (Fuchs et al., 2001).

According to the “Simple View of Reading” (SVR) model (Gough and Tunmer, 1986; Hoover and Gough, 1990; Tunmer and Chapman, 2012), reading comprehension involves decoding skills (hereafter named “word reading skills” as in Hoover and Gough’s (1990) model), listening comprehension skills and the interaction between these two skills (Keenan et al., 2008). This model has mainly been tested with typical readers (children and adults; see the metanalysis by García and Cain, 2014) and more rarely with readers with dyslexia, especially adults. It is nevertheless an interesting starting point for understanding the processes involved in text reading comprehension in adults with dyslexia, who are exposed to clearly established severe and persistent difficulties at the level of the word reading processes. In this context, some studies have attempted to identify some of the factors explaining text reading comprehension performance (for example in terms of visual word recognition, decoding, general knowledge and working memory) in adults with dyslexia (see, for instance, Ransby and Swanson, 2003). However, such studies are very rare and provide only scattered, disparate data which do not allow us to come to a clear and satisfactory picture or interpretation of the processes involved.

The overall aim of this study is therefore to gain a better understanding of the text reading comprehension processes in university students with dyslexia compared to those mobilized by adult skilled readers. More specifically, since graduating at university involves intensive exposure to long and complex texts, this study also aims to investigate the relationship between text reading fluency processes (a more appropriate measure of adult reading ability) and those involved in text reading comprehension.

### Text Reading Fluency in Adults With Dyslexia

A recent study pointed out that text reading fluency provides a more natural and ecological way of assessing reading than word reading fluency (see Rouweler et al., 2020) because words are almost never read in isolation. Despite this, researchers in English-speaking countries do not prefer to measure text reading fluency when assessing dyslexia, because they feel that the text contents may obscure the measurement of word decoding skills. Words in context are indeed read faster than words out of context, because the context can be used as a top-down predictor (Jenkins et al., 2003). This means that readers with poor word reading skills can use contextual cues as a compensatory mechanism to mask their difficulties. Measurements of text reading fluency may therefore appear to be an interesting indicator of the efficiency of adult dyslexic reading skills. Text reading fluency (hereafter TRF) is a complex skill that likely depends on the simultaneous integration of multiple cognitive and linguistic processes (Fuchs et al., 2001). The current conception of TRF takes account of and integrates the ability to group words into syntactic and semantic units as well as the ability to use punctuation to modulate phrasing and intonation while reading (Veenendaal et al., 2014; Paige et al., 2017; Godde et al., 2021). Efficient TRF is behaviorally defined as “*accurate, rapid, effortless reading with appropriate prosody*” (Wolf and Katzir-Cohen, 2001). Its most widely used and accepted measure consists of a time-limited text reading aloud task (Fuchs et al., 2001).

Very recently, a comprehensive meta-analysis conducted by Reis et al. (2020) including 178 studies compared the reading performance of adult readers with and without dyslexia and reported that deficits in TRF are persistent in adults with dyslexia and are expressed by a very large (and significant) effect size (*d* = 1.76). Although the TRF deficit is well established in adults with dyslexia (see also, for example, Callens et al., 2012), only very few studies have looked at the predictors of TRF in adults with dyslexia. To our knowledge, the study by Ransby and Swanson (2003) is one rare work that has directly addressed this issue. In this study, TRF performance was assessed with a composite score from both the Gray Oral Reading Test (GORT-3; Wiederholt and Bryant, 1992), in which a text reading aloud task was followed by a comprehension questionnaire, and the Fast Reading Subtest of the Stanford Diagnostic Reading Test (SDRT, Karlsen et al., 1984). In this subtest, participants were given 3 min (180 s) to silently read one-page stories. Interspersed throughout the stories were 30 highlighted lines, each containing three words. Participants had to choose (in a multiple-choice context) the word that made the most sense. The composite score calculated on the basis of performance on the GORT Reading Aloud Subtest and the SDRT Fast Reading Subtest was called the Reading Comprehension Fluency score. In this study, a wide range of tasks were administered assessing phonological skills (pseudoword reading, phoneme deletion and counting), word recognition, naming speed, vocabulary, listening comprehension (oral versions of the passages from Form B of the *GORT*), verbal working memory (VWM) [assessed with a listening span task derived from Daneman and Carpenter (1980) and a semantic association task], general knowledge and non-verbal intelligence (Raven Progressive Matrices, Raven and Summers, 1986). In all the administered tasks, the performances of the adults in the dyslexia group were significantly lower than those of the control group (except for the non-verbal intelligence test and one phoneme task, namely the phoneme deletion task). Using hierarchical regression modeling, the authors reported that three scores predicted independent variance in text reading fluency (in this case, for comprehension), namely verbal working memory/non-verbal intelligence, phonological processing (a composite score including pseudoword reading, phonemic awareness) and listening comprehension. Interestingly, higher-order factors (such as listening comprehension) explained significantly more additional variance than lower-order factors (such as phonological factors) and there was no indication of any interaction with the group factor. These results suggest that when adults with dyslexia read texts aloud for comprehension, the explanatory factors are the same as those at work in control readers and, as might be expected, higher-order skills have a greater explanatory power than lower-order skills. Using the Gray Oral Reading Test (GORT-3; Wiederholt and Bryant, 1992) to assess TRF in adolescents with dyslexia, Rose and Rouhani (2012) reported that word recognition, verbal working memory and expressive vocabulary (vocabulary subtest of the WISC, Wechsler, 1997) were significant predictors of their scores. A significant interaction between verbal working memory and expressive vocabulary also predicted the TRF scores, with verbal working memory being more involved when adolescents exhibited poor vocabulary, suggesting a compensatory effect of vocabulary skills (higher-order factor).

Although interesting, these two studies do not provide a “pure” measure of TRF, i.e., text reading fluency independently of the reading comprehension process (involving semantic/interpretation processes). Such a measure might unambiguously explain, for example, the influence of higher-order skills such as listening comprehension, which has conventionally been used to explain text reading comprehension scores (Keenan et al., 2008). Thus, one of the objectives of this study is to clarify this point because, based on the SVR framework (Gough and Tunmer, 1986), it has long been considered that both decoding and word recognition skills [assessed by a pseudoword reading fluency (PWF) task and a word reading fluency (WRF) task, respectively] are sufficient to explain TRF performances without any need for recourse to higher-order factors (LaBerge and Samuels, 1974; Allington, 1983; Torgesen et al., 1999; Fuchs et al., 2001; Adolf et al., 2006; Hudson et al., 2008).

Even though they used many different measures, Ransby and Swanson (2003) did not study the role of spelling skills when explaining TRF scores, although research on children and adolescent with dyslexia has shown a reciprocal relationship between decoding and visual word recognition skills, on the one hand, and spelling skills on the other (Berninger et al., 2008; Bazen et al., 2020). Furthermore, the involvement of spelling skills in the reading of adults with dyslexia has been little studied, probably because research on this point has shown a persistent deficit in these skills. For example, the meta-analysis by Reis et al. (2020) reported an impairment among these readers, with the spelling tasks used to assess the ability to spell in this population, such as writing words or pseudowords from dictation (deficit assessed with a Cohen’s *d* of 1.7), making use of the conventions of letter-sound relationships. This result is not surprising since spelling tasks require participants to use orthographic knowledge, the acquisition of which depends partially on phonological factors widely impaired among readers with dyslexia (in children, Manis et al., 1993; Vellutino et al., 2004; but also in adults, Bruck, 1990).

However, some studies suggest that orthographic knowledge/skills in adults with dyslexia might be less impaired than in children. For example, Miller-Shaul (2005) used a variety of orthographic tasks (for example, the orthographic decision task in which participants have to decide whether two orally presented words are written with the same letters or not) and reported some particularly informative results. In this study, the orthographic skills of dyslexics were compared to those of typical readers in two groups of participants, i.e., children in their fourth year of primary school and adult university students. Overall, the performances of dyslexic readers were significantly better in adults than in children, suggesting an improvement in orthographic skills during development, and no difference was observed between the dyslexic and the typical readers in adulthood.

In one of the very few studies to have examined the role of spelling skills and TRF in compensated and non-compensated dyslexic adults, Lefly and Pennington (1991) reported that compensated dyslexic adults performed better than non-compensated dyslexic adults on TRF (as assessed with the GORT test) and spelling tasks. Interestingly, Leinonen et al. (2001) used a text reading aloud task followed by comprehension questions and concluded that advanced spelling skills might help some adult dyslexic readers to compensate for their phonological deficits. This hypothesis is consistent with the results of the study by Siegel et al. (1995), which made use of an orthographic awareness task (designed to measure awareness of the properties of English words and the probable sequence and positions of letters) and revealed that the scores of dyslexic readers from first to eighth grade were significantly higher than those of control readers. The authors suggest that “the difficulties with phonological processing and the increased orthographic knowledge of the dyslexic readers may indicate a reading strategy that relies more on the visual than the phonological features of words.” Overall, these studies indirectly suggest that spelling skills may in some way be used in the TRF skills of adults with dyslexia through one of their components, namely orthographic knowledge, given that these participants’ visuo-spatial memory skills, on which the orthographic coding of words partly relies, seem to be preserved (see the meta-analysis by Swanson and Hsieh, 2009). To summarize, the main predictive factors of a “pure” measure of TRF which would not require extensive semantic processing (contrary to that proposed by Ransby and Swanson, 2003) would primarily consist of lower-order skills, including visual word recognition, decoding and spelling skills, whereas higher-order factors would exert less influence in skilled readers (who may rely on automatized word reading processes) than in individuals with dyslexia.

### Text Reading Comprehension in Adults With Dyslexia

Reading comprehension is a complex cognitive activity that involves “*performing in a very short time a set of operations ranging from the recognition of written words to the construction of a coherent representation of the situation described, through syntactic analysis and the linking of referents and ideas stated in successive sentences*” (Bianco, 2015). Word recognition skills, language and general knowledge activation, working memory and reasoning skills as well as inference-making abilities are involved and often interact during reading comprehension in order to construct a coherent mental representation of the text (i.e., a model of the situation) that integrates the information contained in the text, the reader’s knowledge and the inferences that he or she has made during the reading of the text (van Dijk and Kintsch, 1983; Kintsch, 1988). In line with this proposal, findings from a meta-analysis by Quinn and Wagner (2018) investigating the relationships among components of reading comprehension in a large sample of children and adolescents (*N* = 1,205,581; 155 studies included) have shown that three cognitive factors best predict reading comprehension performance, especially for adolescent readers. These factors include (1) a “decoding” factor corresponding to WRF, TRF and word reading accuracy, (2) a “linguistic comprehension” factor corresponding to general knowledge, semantic and morphological knowledge and listening comprehension, and (3) a “cognitive” factor corresponding to working memory, reasoning and inference-making.

According to this general framework, each of the postulated skills/knowledge involved in reading comprehension may represent potential sources of difficulty for individuals with dyslexia in understanding written text. Interestingly, it seems possible that some of these components may also act as compensatory factors, thereby explaining that whereas some meta-analyses report significantly poorer performance in reading comprehension (Reis et al., 2020), the amplitude of the deficits appears to be much less than that observed for low levels of reading (effect size for visual word recognition is *d* = 1.81; for decoding skills *d* = 2.03 and for text reading comprehension *d* = 0.729). Thus, as mentioned earlier, a number of studies report similar text reading comprehension performance in adults with dyslexia and in skilled control readers when no time constraints are imposed (Lesaux et al., 2006; Parrila et al., 2007; Deacon et al., 2012).

Few studies have systematically investigated the factors (from reading skills to executive functions, general knowledge and listening comprehension skills) that explain text reading comprehension in adult readers with and without dyslexia. The study by Ransby and Swanson (2003) is one of the most comprehensive in this respect. Using hierarchical regression modeling in both adults with and without dyslexia, the authors reported that phonological processing, naming speed, vocabulary, general knowledge, and listening comprehension are good predictors of text reading comprehension. However, one interesting finding was that the predictive power of higher-order factors was much greater than that of lower-order factors, with the respective contributions being similar in both groups of readers. These results are in line with those of the meta-analysis by García and Cain (2014), which reported that the relationship between reading comprehension and oral language comprehension becomes stronger as the reader’s decoding skills become more automatized (see also Verhoeven and van Leeuwe, 2012; Foorman et al., 2015). Consequently, the primary demand faced by most skilled adult readers is not word decoding but instead comes from the nature of the text itself, for example in terms of content and vocabulary complexity (Braze et al., 2007). Therefore, skilled adult readers would be more likely to place greater reliance on listening comprehension and semantics (i.e., vocabulary skills) in support of reading comprehension (Lervåg et al., 2018).

In the study by Ransby and Swanson (2003), VWM and non-verbal intelligence were no longer reported as predictors of text reading comprehension scores once naming speed was included in the analysis. Using Structural Equation Modeling in skilled adult readers, Georgiou and Das (2016, 2018) also reported no influence of VWM capacity (i.e., as assessed with listening span and digit memory) on text reading comprehension scores. However, it can be hypothesized that the tasks used to assess VWM might not have been sensitive enough for the effects to be clearly demonstrated. Working Memory is a limited-capacity memory system that is involved in the temporary storage and processing of information by maintaining, integrating and manipulating information from a variety of sources (Smith-Spark and Fisk, 2007). A number of different span tasks have been developed and one of those to have attracted the most attention from researchers is the reading span task developed by Daneman and Carpenter (1980), which has been argued to provide a good overall measure of the WM capacity involved in reading (that is, the capacity which mobilizes the processes involved in reading comprehension, Daneman and Carpenter, 1980; Friedman and Miyake, 2004; Conway et al., 2005; Smith-Spark and Fisk, 2007). A recent meta-analysis by Reis et al. (2020) reported a VWM deficit for adults with dyslexia, with an effect size of *d* = 0.9, and Ransby and Swanson (2003) found a similar involvement of VWM when explaining text reading comprehension in adults with dyslexia compared to control readers.

### Text Reading Fluency and Reading Comprehension

As mentioned earlier, university students with dyslexia have to read large numbers of long and complex texts. TRF would therefore be a more ecological and appropriate measure of their basic reading competence and may be considered to underlie their text reading comprehension processing, which involves word access and a word-to-text integration process (Perfetti and Stafura, 2014).

Within the general information processing framework, Georgiou and Das (2014) used two indicators of reading fluency, namely fluency at word level and fluency at passage level, to address the question of how reading fluency and text reading comprehension may be related. In their formal framework (Georgiou and Das, 2014), TRF first makes it necessary to identify the isolated words in the text (based on orthographic and phonological processes) and to memorize the sequence of words they belong to. Both these steps are mainly performed under the control of sequential (or) successive processes. Simultaneous processes then come into play as it becomes necessary to process the relationship between words and integrate them into complete units of information (sentences, for example), for example when it is necessary to analyze and synthesize grammatical relationships during reading comprehension. In their revised framework (Georgiou and Das, 2014), simultaneous processing is assumed to predict reading comprehension through the effects of TRF and successive processing is assumed to predict reading comprehension through the effects of word-reading fluency. In addition, simultaneous processing is expected to have a direct effect on reading comprehension because the full, integrated comprehension of the main and subsidiary ideas is required only for a fraction of the text in any given passage. The authors used structural equation modeling (i.e., path analysis) for a sample of 128 university students and showed that successive processing predicted reading comprehension indirectly via text- and word-reading fluency, whereas simultaneous processing predicted reading comprehension both directly and indirectly via text-reading fluency. In a second study, they compared a sample of university students with (*n* = 20) and without (*n* = 23) reading difficulties and showed that the cognitive difficulties experienced by the group of university students with reading difficulties related primarily to successive processing (25% of the sample), and also found that 30% had a dual simultaneous/successive deficit and that only 5% exhibited a simultaneous deficit. The path analysis was not tested because of sample size issues.

In the light of the results of the study by Ransby and Swanson (2003), Georgiou and Das (2014) did not include the TRF measures in their analysis of reading comprehension measures and the TRF measure itself was not a “pure” one, unlike that used by Georgiou and Das (2014), it can be assumed that, in skilled readers, written word recognition (or word reading fluency) and TRF abilities are sufficiently developed to support text reading comprehension. As far as individuals with dyslexia are concerned, two alternative hypotheses can be considered. The first assumes that both word reading and TRF skills are qualitatively too poorly developed to significantly assist text reading comprehension. This hypothesis is supported by the results of Gelbar et al. (2016) reporting that TRF was not a significant predictor of text reading comprehension in secondary students with dyslexia. These results are in line with those showing a dissociation between TRF and text reading comprehension skills in university students with dyslexia (Murray and Wren, 2003; Corkett et al., 2006; Deacon et al., 2006; Cavalli et al., 2019). According to Tunmer and Greaney (2010) and Gelbar et al. (2016), readers with dyslexia would have developed some reading comprehension compensation strategies above the “word” level, thus explaining why some individuals with dyslexia demonstrate age-appropriate reading comprehension abilities that are not explained by their word reading skills and decoding abilities. An alternative interpretation, proposed by Pedersen et al. (2016), suggests that many dyslexics in higher education tend to focus their attention on one subcomponent of the reading process, for example, decoding or comprehension, because engaging in both simultaneously may be too demanding for them. The second hypothesis is that the skills involved in written word recognition and the successive processing of information are too deficient to influence comprehension. However, because TRF is thought to rely on both relatively preserved simultaneous processing (integrating words into whole units of information) and compensatory processes (higher-order factors such as general knowledge, listening comprehension, for example), it may be involved in text reading comprehension.

### The Current Study

The objective of our research is twofold. Firstly, we will compare the text reading fluency and text reading comprehension skills of French dyslexic university students reading in a more transparent orthographic system than English (which is over-represented in the studies cited) on the basis of tests specifically created or adapted for an adult population (listening comprehension, text reading comprehension, TRF, word reading, decoding, phoneme awareness, spelling, visual span, reading span) and on the basis of more general tests that are already available (vocabulary, non-verbal reasoning, general knowledge tests). In line with the literature, we predicted lower scores in the dyslexic group on all the lower-order skills, including word reading fluency, decoding, phoneme awareness, as well as spelling and TRF. Moreover, based on persistent deficits in decoding and visual word recognition skills as well as in VWM (Reis et al., 2020) in adults with dyslexia, we expected reading span to be impaired in this population. In contrast, we expected text reading comprehension performance to be preserved (as it is assessed with no time pressure) in the same way as higher-order skills such as general knowledge, vocabulary and non-verbal intelligence. Finally, based on the meta-analysis by Swanson and Hsieh (2009), we also expected visuo-spatial skills to be preserved in adults with dyslexia.

Secondly, using multiplicative linear regression analysis we will identify the best predictors of both TRF and text reading comprehension in these two populations. To this end, we will test the hypothesis that TRF and text reading comprehension in adults with dyslexia are mediated not only by low-level skills, but also by higher-level skills. We also formulated two alternative hypotheses which contrast the involvement of TRF in text reading comprehension in adults with dyslexia with the case of skilled readers, for whom TRF is expected to be a significant predictor of text reading comprehension.

## Materials and Methods

### Participants

The experiment was conducted in accordance with the Declaration of Helsinki and with the understanding and written consent of all the participants. The project was approved by the local ethics committee (Aix-Marseille University, Marseille, France). One hundred and seventeen participants were recruited (54 adults with dyslexia, DYS; 63 skilled adult readers, SR). All were university students, French native speakers, and had normal or corrected-to-normal vision. The 65% of the participants were enrolled in social science programs (e.g., psychology, law, economics, or archaeology) and 35% were enrolled in science programs (e.g., neurosciences, pharmacy, medicine, chemical physics, or mathematics). The data of four participants (one participant with dyslexia and three skilled readers) were removed from the data set because they performed under the 75th percentile in non-verbal intellectual quotient (IQ) (Raven’s Matrices; Raven et al., 1998). The remaining 113 participants (53 DYS and 60 SR) had a non-verbal IQ within the normal range (above the 75th percentile). None of them reported any neurological or psychiatric disorders. All participants with dyslexia reported major difficulties in learning to read during childhood and had received a formal diagnosis of dyslexia (mean age of diagnosis = 9.17, sd = 3.3) established by a physician in a reference center for learning disabilities. They were recruited at Aix-Marseille University and Lyon University, primarily through the University Disability Service.

As reported in Table 1, the two groups were matched on chronological age, educational level, vocabulary knowledge (the EVIP scale; Dunn et al., 1993), and non-verbal IQ (Raven’s matrices; Raven et al., 1995).

**TABLE 1:** Cognitive profiles of readers with and without dyslexia.

	Readers with dyslexia	Skilled readers	*t*-values		Cohen’s *d*
Chronological age	20.4 (1.9)	20.2 (1.7)	0.09	ns	0.09
Years of higher education	2.43 (1.56)	2.37 (1.69)	0.33	ns	0.04
Non-verbal IQ (raw scores)	43.57 (6.88)	44.84 (5.55)	–1.06	ns	0.20
Visuo-spatial span	6.64 (1.52)	5.96 (1.73)	2.17	*	0.42
**Reading and spelling skills**					
Alouette (efficiency)	119.19 (24.42)	171.73 (24.61)	–11.37	***	2.14
Word reading	76.94 (21.84)	105.5 (22.33)	–6.86	***	1.29
Pseudoword reading	75.71 (28.21)	137.26 (29.89)	–11.25	***	2.11
Text reading fluency	142.67 (29.51)	197.83 (30.54)	–9.70	***	1.83
Reading span	38.3 (8)	44.19 (7.29)	–4.04	***	0.77
Spelling	68.87 (6.02)	75.15 (2.85)	–6.94	***	1.36
**Phonological skills**					
Phonemic awareness (efficiency)	1.18 (0.42)	2.01 (0.45)	–9.81	***	1.89
Phonological short-term memory	4.36 (0.88)	4.93 (0.8)	–3.61	***	0.69
**Comprehension skills**					
Listening comprehension	10.3 (4.25)	10.48 (3.6)	–0.24	ns	0.05
Text reading comprehension	23.23 (5.5)	20.75 (5.8)	2.32	*	0.44
General knowledge	11.43 (3.6)	12.37 (4.1)	–1.28	ns	0.24
Vocabulary knowledge	38.44 (6.23)	40.18 (4.59)	–1.62	ns	0.32

Standard deviations are reported in parentheses. T-values were obtained from unpaired Student t-tests comparing the two groups of participants (***p < 0.001; *p < 0.05;

*^ns^p > 0.10).*

### Material

We administered a battery of 14 tasks to each participant. Administration of the tasks took about 2 to 2.5 h and the tasks were presented in the same order for each participant.

#### One-Minute Word Reading (Word Reading Fluency)

Participants were instructed to read written words aloud as fast and accurately as possible for 1 min. Words were presented on a printed sheet containing six words per line. The 120 disyllabic French words with a length between 4 and 9 letters (mean = 6.4; sd = 1.29) and a frequency varying from low to high (mean = 28.6; sd = 43.4) were selected using the *lexique.org* database (New et al., 2001). An efficiency score which took account of both accuracy (A) and reading time (RT) was then computed for each participant: (A/RT)*60.

#### Two-Minute Pseudoword Reading (Decoding)

Participants were instructed to read 116 written pseudowords aloud as fast and accurately as possible for 2 min. Pseudowords were presented on a printed sheet containing six pseudowords per line. They were one or two syllables in length and had an average letter length of 5.5 (sd = 0.5). Efficiency scores were again calculated for each participant: (A/RT)*120.

#### Text Reading Fluency

Participants were instructed to read a text aloud as fast and accurately as possible in 1 min and to respect the punctuation marks while doing so. The text was taken from *“The red silk scarf”* (Leblanc, 1913), a short narrative literary French text consisting of 434 words and 24 sentences. For the purposes of our task, we reduced the text length by presenting only the first 337 words (17 sentences). The main linguistic characteristics of the text were determined using the *Cordial Neo* software (Synapse Développement, 2019). The sentences in the text were of normal length (mean: 19.8 words/sentence) and had a simple grammatical structure (few adjectives and pronouns, 11.2% and 5.9%, respectively). Moreover, the text included a high proportion of very frequent words [85.8% according to Gougenheim’s Fundamental French (Gougenheim, 1977) and 78.9% according to Dubois–Buyse’s scale (Dubois and Buyse, 1940/1952)], and a limited number of low-frequency words (1.2%). Thus, the readability index of the passage (Flesch score) was equal to 49 on a scale ranging from 1 (very difficult) to 100 (easy), situating it as a text of average complexity (secondary education level). Gougenheim’s Fundamental French (Gougenheim, 1958, revised in 1977) is a list of the 3,500 most common words and of the most usual grammatical concepts in French. The Dubois–Buyse scale (Dubois and Buyse, 1940, revised in 1988) is a corpus of 3,787 commonly used words that are assumed to be known by any French-speaking adult (80% after 6 years of schooling).

#### Alouette

The Alouette test (Lefavrais, 1967) requires participants to read a 265-word text aloud as rapidly and as accurately as possible within a maximum of 3 min. The specificity of this test is that the text consists of real words contained in meaningless but grammatically and syntactically correct sentences, thus preventing dyslexic readers and poor readers from compensating for their written word recognition difficulties by using contextual information. The test yields measures of accuracy (A, number of words correctly read), reading time (RT, time taken to read the text), and reading efficiency [called CTL, computed using the following formula: CTL = (A/RT)*180, where A = accuracy (self-corrections included), and RT = reading time (maximum = 180 s); see Bruyer and Brysbaert (2011), Cavalli et al. (2018), for a detailed presentation of efficiency scores]. Interestingly, the test is standardized for children aged 5 to 14 and provides a score expressed in terms of reading age. The test has now also been standardized for adults with and without dyslexia (Cavalli et al., 2018). The psychometric qualities of this test have been demonstrated in a number of previous studies in both children (Bertrand et al., 2010) and adults (Cavalli et al., 2018).

#### Spelling

In this computerized timed-test, participants were instructed to write down the words they heard as accurately as possible on a sheet of paper (see Tops et al., 2012 for more details). They had 3 s to write down a given word before hearing the next one and were instructed to go on to the next if they could not write the word. They were also warned that they could not go back to a word to correct its spelling. Eighty words were selected from the *lexique.org* database (New et al., 2001). Words varied in spelling consistency (half consistent words, half inconsistent words), written frequency (mean = 47; sd = 100), were from 3 to 8 letters long (mean = 6; sd = 1,35) and were composed of 1 to 2 syllables. Words had been recorded in a soundproof room by a French native speaker prior to the test. Word order was randomized. The final score corresponded to the number of correctly written words (maximum 80).

#### Phonemic Awareness

In this computerized test, participants were instructed to repeat, as fast and accurately as possible, the pseudowords they heard after deleting the first phoneme (e.g., they heard/blə/ and had to say/lə/). The 30 monosyllabic pseudowords with a Consonant-Consonant-Vowel (CCV) structure were selected. Pseudowords were used in order to avoid the activation of lexical knowledge. Reliability (Cronbach’s α) was 0.88 (95% confidence interval [0.83; −0.90]). As in the previous tasks, the final scores were efficiency scores which took account of both accuracy and response times: (A/RT)*100.

#### Phonological Short-Term Memory

This computerized task was selected from the EVALEC battery (Sprenger-Charolles et al., 2005). Participants heard 24 pseudowords which they had to repeat. The length of the pseudowords increased progressively (from 3 to 6 syllables, six items per condition). The task started with a practice session of three items (not included in the final scores). The final scores were efficiency scores: (A/RT)*100.

#### Non-verbal Intellectual Quotient

Non-verbal reasoning abilities were determined using the Standard Progressive Matrices (Raven et al., 1998). The final score corresponded to the number of correctly completed patterns (maximum = 60).

#### Vocabulary Knowledge

We used a short computerized presentation of the French adaptation of the *Peabody Picture Vocabulary Test* (EVIP; Dunn et al., 1993). This task assesses the participants’ receptive vocabulary. The task started with a practice session of four items (not included in the final scores). Only accuracy was recorded (the number of correctly identified words; maximum = 51).

#### General Knowledge

This task corresponds to the subtest of the Wechsler Adult Intelligence Scale (WAIS, Wechsler, 1981). The task was untimed and consisted of 24 questions assessing non-specific general knowledge. The final score corresponded to the correct number of responses (max = 24).

#### Text Reading Comprehension

We created a text comprehension task to evaluate literal comprehension and two types of inferential comprehension skills. We assessed, on the one hand, “text-connecting” inference skills, which require participants to integrate text information in order to establish local cohesiveness and, on the other, “knowledge-based” inference skills, which make it possible to establish links between the text content and the reader’s personal knowledge. Participants had to read three short texts to themselves without time constraints. All three texts were newspaper articles from *Le Monde* concerning the Great Barrier Reef. This topic was chosen to avoid any advantage due to knowledge of the field of study on the part of participants. After reading the texts, participants had to answer eight questions evaluating their comprehension: four questions about explicit literal comprehension and four inferential questions about the comprehension of the implicit information in the texts (two examining text-connecting inferences and two examining knowledge-based inferences). One half of the questions were multiple choice questions, the other half were open questions. Participants were not allowed to refer to the text when answering the questions. The main characteristics of each text are presented in the Appendix Table A1. Reliability (Cronbach’s α) was 0.78 (95% confidence interval [0.74; 0.83]).

#### Listening Comprehension

In this task, participants had to listen to a short story while trying to remember it in order to answer questions. The selected story was a passage taken from the French version of Planet of The Apes (Boulle, 1963). It consisted of 278 words and 22 sentences (mean length: 12.6 words/sentence). The linguistic characteristics of the text were determined using the *Cordial Neo* software (Synapse Développement, 2019). A high proportion of common words were used, thus making the text easy to understand: 84.9% of words belonged to Gougenheim’s core French corpus (Gougenheim, 1977), and 79.5% to Dubois–Buyse’s scale (Dubois and Buyse, 1940; Ters et al., 1988). The story was about a man captured and made prisoner by apes, making the situation incongruous. The understanding of this text requires both precise literal understanding and good inferential reasoning. The story was recorded in a soundproof room by a French-native female speaker. At the end of the story, participants had to answer 20 open questions (10 questions examining literal comprehension, 10 questions examining inferential comprehension). The final score corresponded to a global comprehension score (/20). Reliability (Cronbach’s α) was 0.69 (95% confidence interval [0.56; 0.76]).

#### Reading Span Test

This (computerized) test was created by Daneman and Carpenter (1980) and uses 60 sentences (out of 100) selected from the French version of Desmette et al. (1995). Participants were instructed to read sentences while memorizing the last word of each and indicating if the sentence was meaningless or not. Sentences were presented in blocks containing 2 to 6 sentences. After each block, participants had to recall all the last words of the sentences in the previous block. The task started with a practice session containing one block of two sentences (not included in the final score). The final score corresponded to the number of correctly recalled words (maximum = 60), regardless of the order in which they were recalled (Friedman and Miyake, 2005).

#### Visuo-Spatial Span

This task corresponded to the Visual Pattern Test (Della Sala et al., 1997) and allowed us to examine the ability to remember static visual patterns. Square matrices were presented to participants for 3 s. The participants were then asked to recall the pattern by shading the appropriate squares on a blank matrix. Matrices of increasing difficulty were presented (from 2 to 15 filled squares, three matrices for each difficulty level). The test stopped when the participants could not correctly recall a pattern. The final score corresponds to the mean difficulty level of the last three correctly recalled patterns, with a maximum score of 15.

## Results

As can be seen in Table 1, the *t*-tests revealed that readers with and without dyslexia did not differ on non-verbal IQ, listening comprehension, general knowledge, or the vocabulary knowledge tasks (all t values between −0.2 and 1.2; Cohen’s *d* ≤ 0.32). Interestingly, readers with dyslexia had a greater visuo-spatial span than skilled readers (*p* < 0.05), even if the associated effect size was relatively low (Cohen’s *d* = 0.42). The group with dyslexia also achieved better text reading comprehension scores than skilled readers (*p* < 0.05), and this was associated with a moderate effect size (Cohen’s *d* = 0.44). However, and as expected, they exhibited poorer performance than skilled readers on the phonological tasks (i.e., phonemic awareness, and phonological short-term memory; all *p*s < 0.001), and this was associated with moderate to large effect sizes (all Cohen’s *d* ≥ 0.69). They also achieved poorer reading and spelling performances than skilled readers on all measures, including the Alouette, word and pseudoword reading, the TRF, reading span, and spelling (all *p*s < 0.001), again with moderate to large associated effect sizes (all Cohen’s *d* ≥ 0.77).

### Text Reading Fluency Among University Students With and Without Dyslexia

**FIGURE 1:**
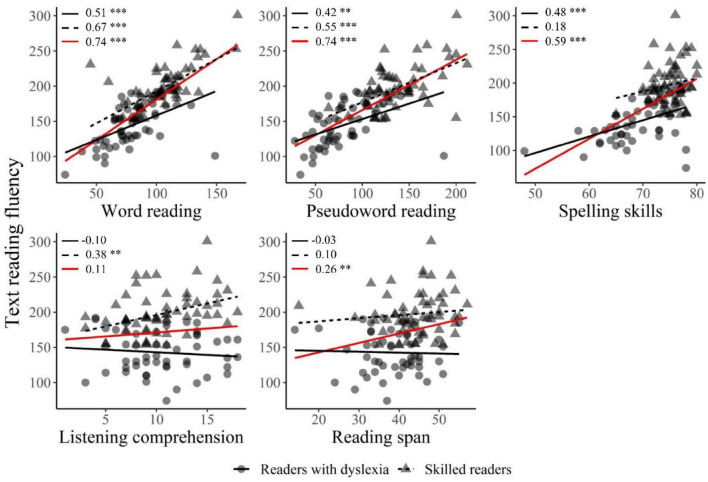
Correlations between TRF and each of the covariates (i.e., word reading, pseudoword reading, spelling skills, listening comprehension, and reading span). Skilled readers are represented by dots, and the corresponding correlation slope is represented by the solid black line. Readers with dyslexia are represented by triangles, and the corresponding correlation slope is represented by the dashed black line. The solid red line represents the correlation slope for the two populations combined. Pearson’s correlations for each slope are indicated in the top left corner of each plot and asterisks represent the significance level of the *p*-value (^**^p < 0.01, ^***^p < 0.001).

In a first step, a correlation analysis was performed between TRF performance and performance on low-level skills (decoding, reading words, spelling) and high-level skills (listening comprehension, vocabulary, general knowledge and reading span). This was done for both groups together, and separately. For reasons of clarity, Figure 1 shows only significant correlations between TRF and each of the covariates we selected in the model of subsequent stepwise regression analysis- for the two populations together (i.e., the red line) and for each group separately (i.e., the black lines). As can be seen, TRF was highly positively correlated with word (DYS: *r* = 0.51, *p* < 0.001; SR: *r* = 0.67, *p* < 0.001; both: *r* = 0.74, *p* < 0.001) and pseudoword (DYS: *r* = 0.42, *p* < 0.01; SR: *r* = 0.55, *p* < 0.001; both: *r* = 0.74, *p* < 0.001) reading skills, both for the two groups separately and when taken together. Spelling skills were positively correlated with TRF when both populations were considered (*r* = 0.59, *p* < 0.001). This correlation was also observed for readers with dyslexia (*r* = 0.48, *p* < 0.001), but not for skilled readers (*r* = 0.18). In contrast, oral comprehension was positively correlated with TRF in skilled readers (*r* = 0.38, *p* < 0.01), but not in readers with dyslexia (*r* = −0.10) or in the two populations taken together (*r* = 0.11). Finally, reading span was also positively correlated with TRF when both populations were considered (*r* = 0.26, *p* < 0.01), but not in each population separately (DYS: *r* = −0.03; SR: *r* = 0.10).

We then applied a multiplicative linear regression model. The selected covariates result from the significant correlations that were observed. Their interactions with the group covariate were also tested (as well as the group covariate itself). A summary of the model is available in Table 2 and the regression coefficients are presented in Table 3. As shown in Table 2, the fifth model fitted the data well (adjusted *R*^2^ = 0.686; RMSE = 22.695) and explained 69.7% of changes in TRF (*R*^2^ = 0.697, *R* = 0.835) based on the combination of pseudoword reading, word reading, and spelling skills, as well as the pseudoword reading * group interaction.

**TABLE 2:** Regression model summary of TRF.

Model	*R*	*R* ^2^	Adjusted *R*^2^	RMSE
1	0.000	0.000	0.000	40.877
2	0.740	0.548	0.544	27.612
3	0.801	0.641	0.634	24.725
4	0.827	0.683	0.674	23.333
5	0.835	0.697	0.686	22.922

**TABLE 3:** Regression coefficients of the model.

Model		Unstandardized	Standard error	Standardized	*t*	*p*
1	(Intercept)	171.673	3.898		44.047	<0.001
2	(Intercept)	94.617	7.231		13.084	<0.001
	Pseudoword reading	0.71	0.062	0.74	11.441	<0.001
3	(Intercept)	64.06	8.697		7.366	<0.001
	Pseudoword reading	0.431	0.077	0.449	5.611	<0.001
	Word reading	0.66	0.125	0.421	5.263	<0.001
4	(Intercept)	82.105	9.508		8.636	<0.001
	Pseudoword reading	0.332	0.077	0.346	4.299	<0.001
	Word reading	0.667	0.118	0.426	5.63	<0.001
	Pseudoword reading * Group	–0.219	0.058	–0.229	–3.76	<0.001
5	(Intercept)	11.153	33.596		0.332	0.741
	Pseudoword reading	0.283	0.079	0.295	3.588	<0.001
	Word reading	0.613	0.119	0.391	5.158	<0.001
	Pseudoword reading * Group	–0.199	0.058	–0.208	–3.434	<0.001
	Spelling skills	1.113	0.506	0.148	2.199	0.03

*T-values and p-values were obtained from a stepwise regression linear model.*

As can be seen in Table 3, reading span and listening comprehension skills do not appear to significantly explain any variability in TRF scores. However, the presence of positive relations in the final model (i.e., model 5) between TRF and pseudoword reading (β = 0.295, *p* < 0.001), word reading (β = 0.391, *p* < 0.001), and spelling skills (β = 0.148, *p* = 0.03) suggests that individuals who had better word and pseudoword reading abilities and spelling skills were also those with better TRF scores. Moreover, the presence of a negative relation between TRF and the pseudoword reading * group interaction (β = −0.208, *p* < 0.001) suggests that pseudoword reading explained TRF to a lesser extent in readers with dyslexia than in skilled readers (see Figure 1).

### Text Reading Comprehension Among University Students With and Without Dyslexia

**FIGURE 2:**
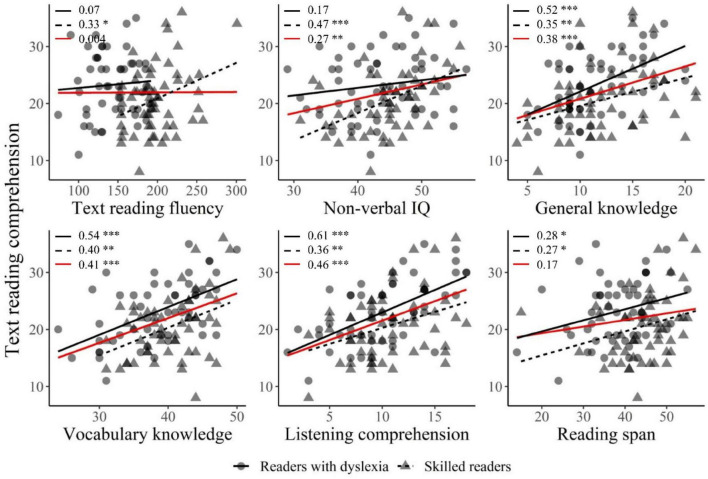
Correlations between text reading comprehension and each of the covariates (i.e., TRF, non-verbal IQ, general knowledge, vocabulary knowledge, listening comprehension, and reading span). Skilled readers are represented by dots, and the corresponding correlation slope is represented by the solid black line. Readers with dyslexia are represented by triangles, and the corresponding correlation slope is represented by the dashed black line. The solid red line represents the correlation slope for the two populations combined. Pearson’s *r* for each correlation slope is indicated in the top left corner of each plot and asterisks represent the significance level of the *p*-value (*p < 0.05, ^**^p < 0.01, ^***^p < 0.001).

A correlation analysis was first performed between Text reading comprehension performance and performance on low-level skills (decoding, reading words, spelling) and high-level skills (listening comprehension, vocabulary, general knowledge and reading span). This was done for both groups together, and separately. For reasons of clarity, Figure 2 shows only significant correlations between Text reading comprehension and each of the covariates we selected in the model of subsequent stepwise regression analysis- for the two populations together (i.e., the red line) and for each group separately (i.e., the black lines). As can be seen, text reading comprehension was highly positively correlated with general knowledge (DYS: *r* = 0.52, *p* < 0.001; SR: *r* = 0.35, *p* < 0.01; both: *r* = 0.38, *p* < 0.001), vocabulary knowledge (DYS: *r* = 0.44, *p* < 0.001; SR: *r* = 0.40, *p* < 0.01; both: *r* = 0.41, *p* < 0.001), and listening comprehension (DYS: *r* = 0.61, *p* < 0.001; SR: *r* = 0.36, *p* < 0.01; both: *r* = 0.46, *p* < 0.001), both when the two populations were taken separately and when they were considered together. TRF was positively correlated with text reading comprehension in skilled readers (*r* = 0.33, *p* < 0.05), but not in readers with dyslexia (*r* = 0.07) or when the two populations were taken together (*r* = 0.004). Non-verbal IQ was positively correlated with text reading comprehension in skilled readers (*r* = 0.47, *p* < 0.001) and when both populations were taken into account (*r* = 0.27, *p* < 0.01), but not in readers with dyslexia considered on their own (*r* = 0.17). Finally, reading span was slightly positively correlated with text reading comprehension in each population separately (DYS: *r* = 0.28, *p* < 0.05; SR: *r* = 0.27, *p* < 0.05), but not when both populations were considered (*r* = 0.17).

We then applied a multiplicative linear regression model. The selected covariates result from the significant correlations that were observed. Their interactions with the group covariate were also tested (as well as the group covariate itself). A summary of the model is available in Table 4 and the regression coefficients are presented in Table 5. As shown in Table 4, the fourth model chosen for the analysis fitted the data well (adjusted *R*^2^ = 0.395; RMSE = 4.485) and explained 41.3% of changes in written comprehension (*R*^2^ = 0.413, *R* = 0.642) based on the combination of listening comprehension, vocabulary knowledge, and the general knowledge * group interaction.

**TABLE 4:** Model summary of text reading comprehension.

Model	*R*	*R* ^2^	Adjusted *R*^2^	RMSE
1	0.000	0.000	0.000	5.766
2	0.488	0.239	0.231	5.056
3	0.578	0.334	0.320	4.754
4	0.642	0.413	0.395	4.485

**TABLE 5:** Regression coefficients of the model.

Model		Unstandardized	Standard error	Standardized	*t*	*p*
1	(Intercept)	21.82	0.56		38.41	<0.001
2	(Intercept)	14.34	1.42		10.10	<0.001
	Listening comprehension	0.71	0.12	0.48	5.62	<0.001
3	(Intercept)	13.29	1.36		9.74	<0.001
	Listening comprehension	0.66	0.12	0.45	5.55	<0.001
	General knowledge * Group	0.28	0.07	0.31	3.77	<0.001
4	(Intercept)	1.99	3.35		0.59	0.553
	Listening comprehension	0.51	0.12	0.34	4.21	<0.001
	General knowledge * Group	0.28	0.07	0.31	4.06	<0.001
	Vocabulary knowledge	0.32	0.08	0.30	3.65	<0.001

*T-values and p-values were obtained from a stepwise regression linear model.*

As can be seen in Table 5, the presence of positive relations in the fourth model between text reading comprehension and listening comprehension (β = 0.349, *p* < 0.001) and vocabulary knowledge (β = 0.301, *p* < 0.001) suggests that individuals who had better oral comprehension and vocabulary knowledge also had better text reading comprehension abilities. In addition, the presence of a positive relation between text reading comprehension and the general knowledge * group interaction (β = 0.314, *p* < 0.001) suggests that general knowledge better explained text reading comprehension performances in readers with dyslexia than in skilled readers (see Figure 2). However, variability in text reading comprehension did not seem to be explained at a significant level by word reading, pseudoword reading, TRF, non-verbal IQ, or reading span.

## Discussion

The overall objective of this study was to gain a better understanding of the text reading comprehension processes in university students with dyslexia compared to those observed in skilled adult readers. To do so, we first compared the text reading fluency and text reading comprehension skills of French dyslexic university students and used a large set of tests to identify their components. We predicted lower scores in the dyslexic group on all the lower-order skills (including word reading fluency, decoding, phonemic awareness, spelling and TRF and reading span) but expected visuo-spatial skills and text reading comprehension scores to be preserved and no different from those of skilled adult readers. We then used stepwise linear regressions to examine the contribution of these skills to, first, TRF skills and, second, to text reading comprehension skills. Our hypotheses were, first, that the main predictive factors of a “pure” measure of TRF would essentially consist of lower-order skills including visual word recognition, decoding and spelling skills, while higher-order factors would have less influence in skilled readers (who may rely on automatized word reading processes) than in individuals with dyslexia. Second, we tested whether low-level skills and higher-level skills would have different impacts on text reading comprehension in adults with or without dyslexia and predicted that the involvement of higher-level skills would play a greater role in dyslexics, due primarily to the difficulties experienced by these participants. We formulated two alternative hypotheses concerning the involvement of TRF in text reading comprehension in adults with dyslexia. The first assumed that (underdeveloped) TRF would not act as a significant predictor of text reading comprehension, whereas the second considered that because TRF appears to rely on both relatively preserved and compensatory processes (higher-order factors such as general knowledge, listening comprehension, for example), it may be involved in text reading comprehension. As far as the skilled readers were concerned, we predicted that TRF skills would provide efficient support for text reading comprehension.

### The Cognitive Profile of Dyslexic University Students

Unsurprisingly, we found that dyslexic university students achieved significant lower performances than skilled readers in phonological tasks (phonemic awareness and phonological short-term memory), reading fluency tasks (Alouette, isolated word and pseudoword reading fluency, TRF) and spelling (word dictation). The largest effect sizes were observed for the Alouette test, pseudoword reading, TRF and phonemic awareness. These results are consistent with studies targeting literacy and phonological skills in adults with dyslexia that have documented persistent deficits in isolated word and pseudoword reading (Ransby and Swanson, 2003; Wolff, 2009; Callens et al., 2012; Swanson, 2012), spelling (Erskine and Seymour, 2005; Parrila et al., 2007; Swanson and Hsieh, 2009; Nergård-Nilssen and Hulme, 2014), TRF (Nergård-Nilssen and Hulme, 2014; Suárez-Coalla and Cuetos, 2015; Reis et al., 2020), phonological skills including phonemic awareness and phonological short-term memory tasks (Ramus et al., 2003; Miller-Shaul, 2005; Lindgrén and Laine, 2011; Swanson, 2012), and reading span (for which a reading task has been used to confirm the results observed with oral tasks, see Reis et al., 2020). The Cohen’s *d* values confirmed those reported in the meta-analysis by Reis et al. (2020), which observed larger effect sizes in reading and spelling tasks as well as in phonological tasks.

We also showed that scores on the listening comprehension, vocabulary, general knowledge (WAIS information), and non-verbal reasoning (Raven’s Matrices) tasks were similar to those of skilled readers. However, Reis et al. (2020) reported very low but significant Cohen’s *d* values on these tasks (vocabulary, *d* = 0.59 and non-verbal IQ = 0.18). Furthermore, Ransby and Swanson (2003) found that adults with dyslexia scored significantly lower than skilled adult readers on vocabulary (receptive and productive) and listening comprehension tasks as well as on general knowledge. The discrepancies between results may be due, at least in part, to the wide variability in the cognitive profiles of dyslexic students. For example, the students in Ransby and Swanson’s study all had special education backgrounds due to their reading disorder, whereas the students in our study all came to university after a conventional school career, which in most cases had also involved support from physiotherapists.

Our results also showed the visual-spatial span of students with dyslexia to be significantly larger than that of skilled readers. These results are in line with those of the meta-analysis by Swanson and Hsieh (2009), which found a trend (also non-significant) in favor of dyslexics. These results may explain why students with dyslexia appear to use significantly more visual-spatial cues than skilled adult readers when they read texts for comprehension (Cavalli et al., 2017). Dyslexic participants were also found to achieve higher scores in the text reading comprehension test (under unconstrained time reading). These surprising findings must be interpretated in the light of those obtained in research showing that dyslexic adults’ text reading comprehension is equivalent to that of their skilled reading peers when they are allowed to read with no time constraints (Miller-Shaul, 2005; Parrila et al., 2007; Tops et al., 2012; Cavalli et al., 2019) as well as of data from the meta-analysis conducted by Reis et al. (2020), which showed that the effect sizes characterizing reading comprehension in dyslexic and skilled adults readers are small in languages with opaque orthographies such as French, and that the differences are reduced when the tests are not performed under time pressure. The absence of time constraints in our text reading comprehension test was undoubtedly beneficial for adults with dyslexia, who are less fluent in reading text and make more errors than their normal reading peers (Pedersen et al., 2016). The ability to read at their own pace for comprehension, reread if needed, and correct errors are all reading strategies that may participate in the comprehension performance of adults with dyslexia (Moojen et al., 2020). Furthermore, the fact that TRF was assessed independently of text reading comprehension likely meant that the cognitive resources involved in text comprehension were less impacted by a low fluency level.

Another reason is the length of the text and its complexity. For example, Ransby and Swanson (2003) used the GORT test in their study. This presents narrative and expository texts which are of average length (between 80 and 150 words) and have a lexical, syntactic and semantic complexity that is considered to be less than that of the texts we used, which were particularly suitable for adults (news articles from the daily newspaper *Le Monde*, intended for adults). One of the consequences of using these texts is that readers may have to draw heavily on their general knowledge to be able to understand precisely what they are currently reading. In line with this interpretation, the results of the regression analyses we conducted show that dyslexic readers made extensive use of this knowledge (see the next section on text reading comprehension) when reading for understanding. Indeed, Keenan et al. (2008) showed that the involvement of high-level factors, such as listening comprehension skills, in reading increases with increasing text length, to the benefit of lower-level factors (e.g., decoding skills).

### Text Reading Fluency in University Students With and Without Dyslexia

The regression model that best fits our data is a three-factor model including word reading, pseudoword reading, and spelling. Explaining 69.7% of the variance in the two populations, it enabled us to identify low-level literacy skills as the best predictors of TRF in both samples. We also reported that decoding skills (as assessed by a pseudoword reading task) explained TRF to a lesser extent in dyslexic readers than in skilled readers. This result is consistent with data from dyslexic adolescents (Rose and Rouhani, 2012) showing that word reading is a stronger predictor of TRF than pseudoword reading in this population. This is no surprise since pseudoword reading scores in our sample were clearly deficient (*d* = 2.11) when compared to word reading scores (*d* = 1.29), a result which is consistent with many other studies (Reis et al., 2020) and which confirms that TRF in dyslexic students probably relies mainly on visual/orthographic word codes due to their phonological deficits, whereas phonological codes would also be involved in skilled readers. This interpretation is in line with that proposed by Siegel et al. (1995), Leinonen et al. (2001) and Miller-Shaul (2005), who suggest that individuals with dyslexia may compensate for their phonological deficiencies when reading by mobilizing less impaired spelling skills (*d* = 1.36). Visual-spatial abilities could in some way support the visual/orthographic abilities activated during word reading (and spelling). However, they would operate indirectly, as shown by our results, since visual-spatial abilities were not clearly identified as a predictor of TRF. Recent findings by Franzen et al. (2021) using eye movement recordings showed that dyslexic adults may use a different visual sampling strategy during text reading. Contrary to our expectations, higher-order factors, such as listening comprehension, vocabulary or general knowledge, did not emerge as significant predictors of TRF in French university students. These results are contradictory to those of Ransby and Swanson (2003) who reported that higher-order factors explain more variance than lower-order factors (see also Rose and Rouhani (2012) with adolescent dyslexics). This difference can be explained by the demands of the tasks in the two studies. While the participants in Ransby and Swanson’s (2003) study were asked to read the text aloud and then answer questions, a task requiring extensive semantic processing that may demand the activation of general knowledge, vocabulary and processing skills involved in listening comprehension, the participants in the TRF task we proposed were not. Finally, using a “pure” TRF task, verbal working memory (in our case measured with the reading span task) does not appear to be a predictor of TRF for either dyslexic readers or skilled readers. These results echo the data from the literature showing that the verbal working memory of dyslexic adults is poorer than that of skilled readers (Hatcher et al., 2002; Ransby and Swanson, 2003; Miller-Shaul, 2005; Swanson et al., 2009; Martinez Perez et al., 2013; Nielsen et al., 2016; Eloranta et al., 2018) and that its relationship to TRF is weak (Peng et al., 2018) and decreases over development (Pham and Hasson, 2014). When concerned with skilled readers, it is possible that the direct link between Working Memory and TRF is not identifiable in our study with the tests we used, but also that this link may not direct but indirect, i.e., mediated by another skill (e.g., processing speed, attentional resources, or general knowledge), as Hannon (2012) suggests.

### Text Reading Comprehension in University Students With and Without Dyslexia

Our best-fitting model of reading comprehension, explaining 41.3% of the variance, is a three-factor model involving listening comprehension, general knowledge and vocabulary. It is consistent with the results reported by Ransby and Swanson (2003) who showed that higher-order factors explained significant variance in both adult dyslexics and skilled readers, thus suggesting that text reading comprehension in this population relies primarily on top-down processes. One difference between this study and our own is that we found that general knowledge explained text reading comprehension scores in dyslexic readers better than in skilled readers, thus suggesting that the former group relies heavily on semantic information (possibly as a compensatory mechanism) when understanding written texts. Surprisingly, and contrary to our expectations, neither word reading, pseudoword reading, TRF nor reading span explained text reading comprehension scores at a significant level. Even more surprisingly, this was also true of skilled readers, for whom Georgiou and Das (2014) found significant effects of these factors. However, in an experiment which was more similar to our own, Ransby and Swanson (2003) found very little additional contribution of lower-level factors such as word reading and decoding (about 5% but significant) to explained variance. With skilled adult readers, Gonçalves et al. (2021) also reported the influence of both high-level factors such as vocabulary and listening comprehension and low-level factors (such as word reading) on explaining text reading comprehension performance although the authors observed a greater explanatory power of the former. In our study, the questions used in the text reading comprehension test are implicit and explicit questions. It is then possible that performance on explicit questions depends on decoding and word reading skills, whereas performance on implicit questions relies on interpretative processes involving high-level knowledge (e.g., general knowledge). We did not perform an analysis taking this parameter into account but this hypothesis should be tested in future work. However, as far as the dyslexic students are concerned, these results are in line with those of Gelbar et al. (2016), who found no significant contribution of TRF in the text reading comprehension scores of adults with dyslexia. The authors suggested that readers with dyslexia might have developed some reading comprehension compensation strategies above the “word” level, thus explaining why some individuals with dyslexia demonstrate age-appropriate reading comprehension abilities that are not explained by their word reading skills and decoding abilities. It is possible to hypothesize that lower levels of the reading process have a much smaller influence on skilled readers reading in a more transparent orthographic system than English. This would be due to the semantic demands of reading long texts, on the one hand, and automatized visual word recognition processes, on the other. Another possibility is to follow the lead given by Duke and Cartwright (2021) and consider that the overlap between the word recognition and listening comprehension components of the SVR model may not be entirely separate processes. In line with this proposal, Perfetti and Stafura (2014) suggest that the lexicon might play a central role in linking the word identification and comprehension systems. This would explain why the involvement of vocabulary knowledge and semantic systems in high-functioning dyslexics appears to compensate for an impaired written word recognition process. In skilled readers faced with long texts adapted to their cognitive level, comprehension processes would be central to successful reading and would take over from lower-order processes.

To conclude, among the important results of this study, we have shown that Text reading fluency and text reading comprehension do not rely on the same abilities in university students with and without dyslexia. While TRF skills in adults with dyslexia are based on the activation of visual/orthographic codes of words (phonological codes are difficult to be activated), skilled readers use orthographic and phonological codes of the words they read in a flexible way. The corollary of these results is that when participants are asked to read aloud a text and are warned that it is not a comprehension task, high-level knowledge is not strongly mobilized. An TRF task therefore appears to be an interesting ecological task for testing the ability to read (and decode) written material at the university that consists of long texts. This is in contrast to research with adults with dyslexia which uses mainly single word or pseudoword reading tasks.

This study also shows that when university students with dyslexia have to understand a text precisely, their answers do not depend on their ability to decode and read words but, and more importantly than for skilled readers, on their general knowledge, This enables them to achieve a level of reading comprehension that will allow them to pursue higher education. This is one compensatory mechanism that needs to be further elucidated in future research providing a better understanding of dyslexic compensated reading.

## Data Availability Statement

The raw data supporting the conclusions of this article will be made available by the authors, without undue reservation.

## Ethics Statement

Ethical review and approval was not required for the study on human participants in accordance with the local legislation and institutional requirements. The patients/participants provided their written informed consent to participate in this study.

## Author Contributions

All authors contributed to the experimental, data analysis, and writing parts of the manuscript.

## Conflict of Interest

The authors declare that the research was conducted in the absence of any commercial or financial relationships that could be construed as a potential conflict of interest.

## Publisher’s Note

All claims expressed in this article are solely those of the authors and do not necessarily represent those of their affiliated organizations, or those of the publisher, the editors and the reviewers. Any product that may be evaluated in this article, or claim that may be made by its manufacturer, is not guaranteed or endorsed by the publisher.

## Footnotes

1 Approximately 1.4% of the student population in France (unpublished data from University Disability services); between 4% and 6.3% in the United Kingdom (*UK Higher Education Statistic Agency*); between 1.6% and 6.4% in Spain (López-Escribano et al., 2018); and between 1.5% and 4% in Sweden (Wolff and Lundberg, 2002).

## Appendix

**APPENDIX TABLE A1:** Main metric characteristics of the selected articles.

	Text 1	Text 2	Text 3
Number of paragraphs	8	7	6
Number of sentences	35	35	45
Number of words	531	465	477
Number of sentences/paragraph	4.4 (3.3)	5 (5.1)	7.5 (2.8)
Number of words/sentence	15.2 (11.5)	13.3 (11.3)	10.6 (8.6)

*Standard deviations are reported in parentheses.*
